# Using responsive feedback from routine monitoring data to guide course corrections for a family planning intervention in Nigeria

**DOI:** 10.12688/gatesopenres.14591.2

**Published:** 2023-11-16

**Authors:** Dominique Meekers, Olaniyi Olutola, Lynn Abu Turk

**Affiliations:** 1 Department of International Health and Sustainable Development, Tulane University School of Public Health and Tropical Medicine, New Orleans, Louisiana, 70112, USA; 2 DKT International, Lagos, Nigeria; 3 Department of Epidemiology and Medical Statistics, University of Ibadan, Ibadan, Oyo, Nigeria; 4 Department of Epidemiology, Tulane University School of Public Health and Tropical Medicine, New Orleans, Louisiana, 70112, USA

**Keywords:** responsive feedback, family planning, routine data, interrupted time series, mass media

## Abstract

**Background:**

This paper aims to promote the use of simple interrupted time series (ITS) analyses of routine data as a responsive feedback tool to improve public health programs. Although advanced ITS techniques exist, their use is often not feasible due to limitations in funding or research capacity. We propose an Excel-based analysis that requires minimal resources or statistical expertise, and illustrate it by measuring the effect of a radio campaign to promote a family planning call center in Nigeria on the demand for family planning information.

**Methods:**

We used a single group interrupted time series design (ITS) as a responsive feedback mechanism to determine whether the radio campaign influenced use of the Honey&Banana call center. ITS is ideal when there is no control group. ITS uses the pre-intervention trend to predict what would have happened if the intervention were absent.

**Results:**

After conducting ITS analyses, the results show that the number of calls requesting family planning information increased throughout the campaign period, with a gain of about 500 additional calls per month, and then decreased after the campaign ended. However, the number of calls gained from the campaign was substantially lower than anticipated.

**Conclusions:**

While end-of-project impact evaluations are necessary, there should be regular feedback system to provide program implementers with information about the status of the project, such as failures, successes, and areas of improvements. This would allow implementers to make necessary adjustments as needed throughout the intervention period. The finding that the radio campaign was not living up to expectations helped Honey&Banana program implementers to end the campaign prematurely and re-allocate resources to a more promising activity. Our research shows that basic Excel-based ITS analysis of routine data can be a useful tool for receiving regular feedback to guide programming improvements for organizations that have limited resources and/or research capacity.

## Introduction

There is growing recognition that many public health programs—as well as broader development initiatives—have been using a rigid one-size-fits-all implementation approach, despite the fact that they are dealing with very complex issues, often in rapidly changing circumstances ( Harvard Kennedy School, 2014; Viswanath *et al.*, 2019). Program implementers typically decide whether to continue or scale up their current approach based on the findings from end-of-project impact evaluations. Although end-of-project evaluations are invaluable for improving the design of future interventions, by definition they are conducted too late to benefit ongoing programs. Even when mid-project impact evaluations are conducted, by the time the results are available, limited time may be left to make course corrections.

To address this issue, scholars and donors alike are increasingly advocating for incorporating responsive feedback mechanisms, sometimes called feedback loops, into the design of public health programs. For example, the Bill and Melinda Gates Foundation has sponsored *The Curve*, a global community of practice that aims to help programs become more skilled at using responsive feedback to achieve better results ( https://the-curve.org), and the journal *Global Health: Policy and Practice* is devoting a special issue to the subject. Responsive feedback mechanisms aim to “…allow intervention information to flow back to the program implementers on an ongoing basis so that appropriate changes in intervention delivery strategy can be adopted throughout the implementation period” ( Pant *et al.*, 2023). As such, responsive feedback serves an important learning function, based on successes as well as failures. Rapid cycles of project planning, obtaining responsive feedback, pause and reflection, and revisions (including replacing activities that do not perform as well as anticipated with more promising ones), are used to drive continuous program improvement ( Harvard Kennedy School, 2014; Viswanath *et al.*, 2019).

A cursory review of projects that applied responsive feedback ( Aisiri, 2019; Ajijola *et al.,* 2023; Ejeh *et al.*, 2021; Onwuasor *et al.*, 2022) shows that a wide range of data collection techniques can be used ( *e.g.,* social media feedback, rapid assessment phone surveys, self-administered survey tools, call-in radio programs, exit interviews at facilities, focus group discussions). The commonality between these techniques for obtaining feedback is that all of them allow frequent data collection. Indeed, frequent data collection is essential for facilitating regular cycles of evidence-based programmatic course corrections. Because many public health programs collect routine monitoring data on a monthly or quarterly basis, they can be an invaluable tool for incorporating responsive feedback into program implementation and revision.

The aim of this paper is to advocate for the use of simple interrupted time series analyses of routine monitoring data as a responsive feedback tool for continuous program improvement, particularly in instances when the use of more sophisticated techniques is not feasible (e.g, due to lack of funding or limited research capacity). We illustrate the approach by examining the effect of a radio campaign to promote a family planning call center in Nigeria on the demand for family planning information and discuss how the findings were used to adjust the program activities.

### Intervention

Honey&Banana (H&B) is a program dedicated to provide family planning information and services, which involves pregnancy prevention, child spacing and family size limitation. It is implemented by DKT International Nigeria (DKT). To receive confidential family planning advice, individuals are encouraged to use the program’s website, social media pages, or its toll-free call center. H&B focuses primarily on modern, long-acting reversible contraceptive methods (LARC). The call center can be reached by calling 55059; all call center agents have been trained to provide confidential information about family planning and ways to access it. If a caller intends to use a family planning method, the agent will refer them to a local family planning clinic from DKT’s network of partner clinics. The call center offers family planning information in five different languages: English, Hausa, Igbo, Yoruba, and Pidgin English.

To promote the call center, H&B developed a large-scale radio jingle campaign that aimed to increase the number of callers requesting family planning information. Because 19% of currently married women and 48% of unmarried sexually active women have an unmet need for family planning ( National Population Commission (NPC) and ICF International, 2019), the latent demand for family planning information is believed to be high. The campaign was diffused on 28 radio stations throughout 12 states (Abia, Abuja FCT, Delta, Edo, Kano, Lagos, Nasarawa, Niger, Ogun, Oyo, Rivers, Sokoto). These states were chosen because of the presence of numerous partner clinics that would be able to handle the anticipated increase in demand for family planning services. The campaign was done by using seven different jingles that encouraged individuals to contact the call center for family planning information or to request a referral to a family planning provider. As such, the jingles aimed to address the latent demand for family planning information (rather than to provide family planning information or education). Given the size of the campaign, a large increase in the volume of calls was anticipated. It was also hoped that awareness of the call center would subsequently spread further through word of mouth.

The radio jingles were broadcast in English, Pidgin, or Hausa, depending on the location, starting in December 2021. The original plan was for the campaign to run for six months, starting in December 2021. However, based on monthly feedback on the number of requests for family planning information received by the call center, the implementers decided to end the campaign in March 2022.

## Methods

### Study design

This paper applies a single group interrupted time series design as a responsive feedback mechanism for assessing whether the *Honey&Banana* radio campaign helped increase use of the call center. Interrupted time series studies have been used to measure the effect of a wide range of public health policies and programs ( Booth *et al.*, 2018; Cheng *et al.*, 2016; Fuseini *et al.*, 2022; Hategeka *et al.*, 2020; Hudson *et al.*, 2019; Humphreys *et al.*, 2017; Ma *et al.*, 2020; Owuor & Amolo 2019; Ramsay *et al.*, 2003; Turner *et al.*, 2020). However, they are typically used as an end-of-intervention impact evaluation ( Bernal *et al.*, 2019), rather than a responsive feedback mechanism.

The most basic form of the ITS design simply examines the overall trend in a key outcome measure. This design is typically used when no control group is available. When the outcome measure is graphed over time, an interruption of the trend is expected to occur after the start of the intervention. This interruption may entail a change in the level of the outcome measure, a change in the rate at which the outcome had been improving, or both ( Bernal *et al.*, 2017). The ITS design then uses the pre-intervention trend in the desired health outcome to predict what likely would have happened in absence of the intervention. In other words, the counterfactual is based on the assumption that the pre-intervention trend would have continued. Comparison of the actual and projected trends gives an estimate of the impact of the intervention. Because this can be done for successive time intervals ( *e.g.* every month or quarter), implementers get continuous feedback about the effectiveness of their intervention.

To use interrupted time series analyses of routine monitoring data as a responsive feedback mechanism, the intervention activity needs to have a clearly defined starting point. Many family planning programs implement social or mass media campaigns ( *e.g.* to encourage contraceptive use) that fit this requirement. Because several pre- and post-intervention data points are needed for the analysis, routine monitoring data ( *e.g.,* the monthly number of family planning clients or the number of contraceptives distributed) that are collected at fixed intervals are an ideal data source.

Our study uses monitoring data on the monthly number of calls received during the pre-campaign period (January to November 2021) to calculate 1) the linear trend in the number of calls, and 2) the logarithmic trend. For simplicity, we estimated the pre-intervention trend with the “trend line” option in Microsoft Excel 365. Although more advanced modeling programs are available, Excel has been used in other studies (see *Turner et al.* (2020)) and has the advantage that the approach can easily be replicated by programs that have limited internal research capacity.

The linear trend assumes there is a steady increase in the number of calls received. The logarithmic trend assumes that the number of calls initially increases fairly rapidly, but then levels off. It has been noted that it is important to consider whether a linear model is appropriate, as certain outcomes may hit a natural ceiling ( Jandoc *et al.*, 2015). Since other sources of family planning information exist, the number of people who are interested in using the call center may eventually hit a ceiling. Hence, in the long term the logarithmic trend may be more realistic. We opted to model both the linear and logarithmic trends and used the relevant equations to project the expected number of calls in absence of the radio campaign. The formulas for the linear and logarithmic equations were used to calculate the projected number of calls for the months from December 2021 through May 2022.

Time series data can be affected by autocorrelation, for example when data points that are close together tend to be more similar than those further apart. Autocorrelation in the pre-intervention data could affect the estimation of the trends that are used to generate the post-intervention counterfactual. Although we have no theoretical reason to suspect the presence of autocorrelation in the pre-intervention data, we heeded the recommendation to test for autocorrelation ( Raadt, 2018). We used R Project software (version 4.2.2) to conduct the Durbin-Watson autocorrelation test. The values of the Durbin-Watson test range from 0 to 4, a value of 2.0 indicating that no autocorrelation is detected. The results of the test confirmed that there is little or no autocorrelation in our pre-intervention data (DW=1.9421; p=0.3112).

For our study, we anticipated that there would not be any substantial lag between the start of the radio jingles and the desired increase in the number of incoming calls requesting family planning information ( Bernal *et al.*, 2017). A lag is unlikely to occur because the radio campaign provides potential callers with an immediate solution for their unmet need for family planning information and because they are most likely to contact the call center while the call center short code is fresh in their memory. Hence, we anticipate observing an increase in the number of calls received, as soon as the campaign started in December 2021. For each of the estimation models (linear and logarithmic ITS), we estimated the effect of the radio campaign by calculating the difference between the actual and projected number of calls received for the period from December 2021 through May 2022.

As the exposure to the radio campaign was not randomized, it is important to acknowledge that other factors, such as different program activities, may have influenced the use of the call center. These variables could possibly lead us to misinterpret how well the radio campaign was working. Since projected trend assumes the pre-intervention trend will continue, it is important to ensure that the pre-intervention trend – including any outliers – is well understood. Prior to the intervention, H&B carried out two brief campaigns which could have increased use of the call center. Between April 1 to May 30, 2021, H&B launched a campaign to encourage use of Sayana Press injectable contraceptives, reaching out to existing users to remind them of their next injection date, and sending an SMS blast to previous users who had not received an injection for over three months. In August 2021, H&B conducted several promotional activities in celebration of the third anniversary of the call center, including intensified activities in affiliated family planning clinics, digital and social media platforms, and a small e-voucher campaign. From February 14 through March 31, 2022, DKT also conducted an HIV self-testing campaign during which self-test kits were distributed to post-secondary students in 29 states. Although this campaign did not focus on family planning, students were advised to contact the *Honey&Banana Connect* call center with any questions or concerns, which may have increased the volume of calls during the radio jingle campaign.

Through the H&B website, DKT's contraceptive products, H&B's social media pages, and the radio campaign, the call center toll-free number is being widely promoted. However, the H&B website was offline from January to November 2021 due to a major attack and was subsequently redesigned. While the restored website was launched at the same time as the radio campaign, it mainly focuses on providing information about contraceptive products. The homepage displays the call center logo and phone number but primarily provides details about the different contraceptive products available, with links to request information about nearby certified family planning clinics and an online store. The toll-free number is also printed on the packaging of most DKT contraceptive brands, such as Fiesta condoms. Regular daily social media posts on Facebook and Instagram also list the toll-free number throughout the study period. As none of these channels were specifically focused on the period from December 2021 to March 2022, it is unlikely that they significantly influenced the results.

There are other potential factors that may have affected the results, such as family planning programs implemented by the Nigerian public sector and other non-governmental organizations. The Society for Family Health and PSI Nigeria are two examples of non-governmental social marketing organizations that work across the country and deliver access to subsidized family planning products and promote their use. The Nigerian Urban Reproductive Health Initiative, implemented by Johns Hopkins Center for Communication Programs, also aims to increase contraceptive use in specific states including Lagos, Kaduna and Oyo. These programs may have affected the increase in demand for family planning information and services, but we are not aware of any programs that were implemented during the diffusion of the radio campaign (December 2021–March 2022). These organizations' communication activities aim to address the demand for family planning information, so they are unlikely to confound our analysis.

### Data source

The data source for this responsive feedback study consists of public records on the number of incoming calls received by the Honey & Banana Connect call center, as published by DKT International Nigeria ( Olutola *et al.*, 2023). The database excludes calls that were not related to family planning ( *e.g.,* wrong number, crank calls, or calls not related to family planning). DKT generated the database based on customer records about all incoming calls received by the call center, which are entered using Customer Relations Management (CRM) software. The number of family planning related calls was extracted from the CRM customer records and aggregated by calendar month for inclusion in the database. Our outcome indicator is the monthly number of incoming calls that were related to family planning. Our study analyzes call center records of the 12,490 incoming calls received by the call center during the period from January 1, 2021 through May 31, 2022. Although using a longer post-intervention period is preferable when there are no known confounding factors (such as other campaigns), this was not feasible due to the start of an H&B e-voucher campaign in June 2022.

The Tulane University Human Research Projection Office determined that this study is not human subjects research as define by the Common Federal Rule and does not require IRB review and approval (Ref #2023-590).

### Study limitations

One of the main limitations of our study, which it shares with all ITS studies, is that it relies on an artificially constructed counterfactual that may not accurately represent what truly would have happened in absence of the intervention. Therefore, the accuracy of the estimated magnitude of the intervention impact depends heavily on the assumption that the pre-intervention trends would have continued. Outliers in the pre-intervention trends, like the ones that were observed in our example, can affect the accuracy of the projected trend. The two outliers in our example resulted from small interventions conducted by H&B itself and that are typical H&B activities. Hence, it is reasonable to assume that the pre-intervention trend would continue. Nevertheless, use of a single group ITS design with routine monitoring data can be a valuable approach to receive responsive feedback about a new intervention is working, even if the exact magnitude of the intervention effect is uncertain.

Another limitation of the ITS design is it cannot easily be used when there are other competing campaigns. Due to the launch of a large-scale e-voucher campaign, we only have two data points after the radio campaign ended, which limited our ability to assess any longer-term effects of the campaign.

## Results

### Trend in requests for family planning information

A number of authors recommend visually inspecting pre-intervention trends for instability and potential outliers prior to ITS analysis ( Biglan *et al.*, 2000; Jandoc *et al.*, 2015). High variability in the pre-intervention time series makes it harder to detect an intervention effect. Outliers may indicate that the ITS assumption that the pre-intervention trend would have continued in absence of the intervention is not valid. Some authors therefore re-analyze the data with the outliers removed ( Hudson *et al.*, 2019).

**Figure 1.**
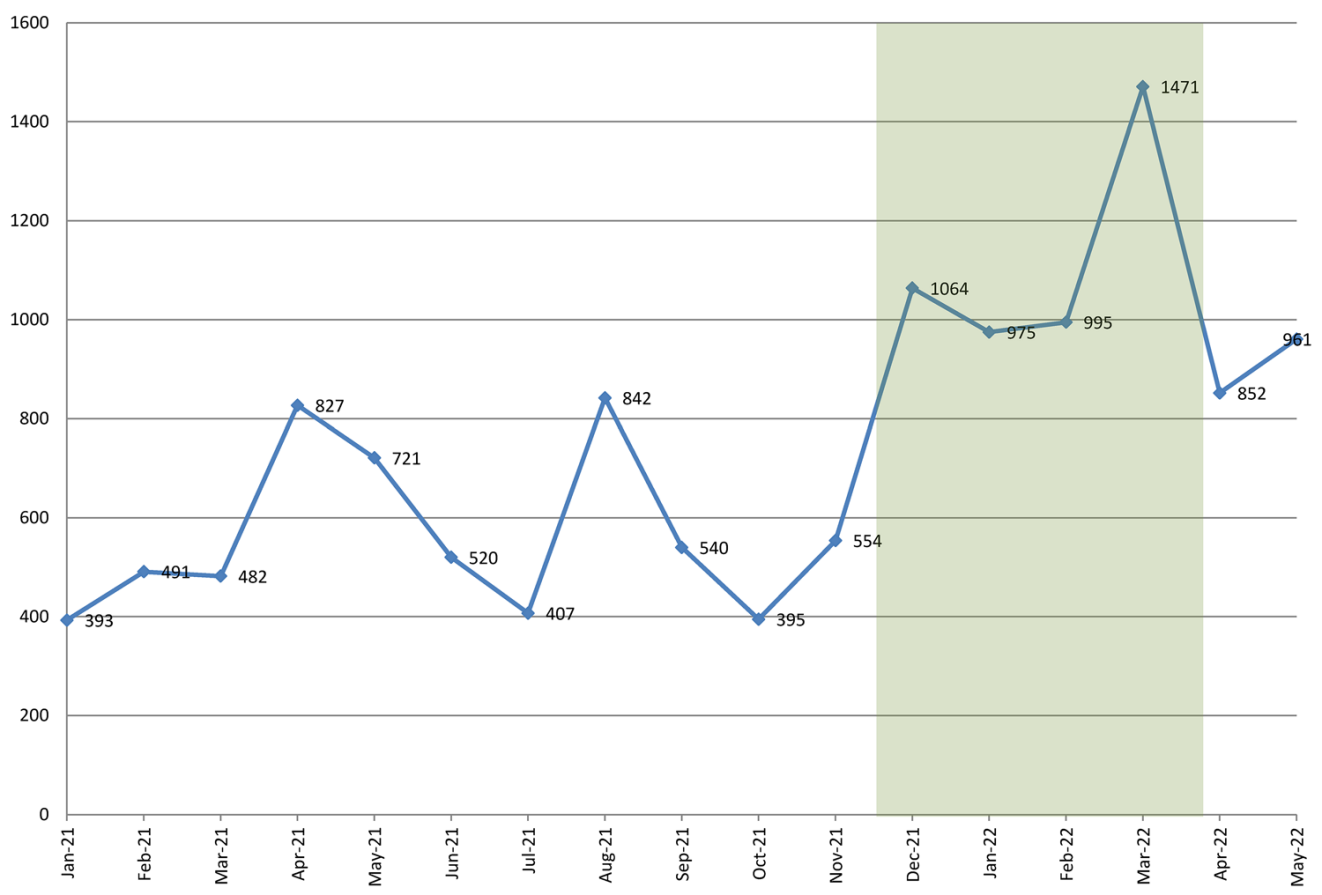
Number of requests for family planning information received. Note: Shaded area depicts the duration of the radio campaign.

Figure 1 shows the observed trend in the number of calls requesting family planning information received by the call center, with the intervention period shaded in green. During the pre-campaign period (from January through November 2021) the monthly number of calls hovered roughly between 400 and 550, except for two notable outliers. The first outlier is the peak in April–May (827 and 721 calls, respectively), which coincides with the period when the program conducted a campaign to promote the use of Sayana Press injectable contraceptives ( Honey&BananaConnect, 2022). The second outlier is the peak of 842 calls in August, which coincides with the third anniversary of the call center. As part of the anniversary celebrations, the program intensified social media promotions of the call center, and implemented a small e-voucher campaign for free contraceptives ( DKT International Nigeria, 2021). The anniversary also was also noted in the press, which may have further increased awareness of the call center ( Brand Spur Media and Marketing Services, 2021; News Wings, 2021). Apart from these two temporary peaks, the monthly number of requests for family planning information showed fairly little variation during the pre-campaign period, and increased only minimally.

Following the start of the radio campaign in December, the number of calls requesting family planning information nearly doubled, from 554 in November to 1,064 in December. It stayed at roughly this level through February (995 calls), and further increased to 1,471 in March. This latter increase may have resulted from the HIV self-testing campaign that was conducted at post-secondary institutions. Recipients of the self-test kits were advised to contact the *Honey&Banana Connect* call center with questions or concerns, which may have increased the volume of calls. By April, when the radio campaign had ended, the number of requests for family planning information decreased, but remained substantially above the pre-campaign levels (852 calls in April; 961 in May).

### Single group interrupted time series (ITS) results

Although the trend shown in Figure 1 clearly indicates that the radio campaign had an effect on use of the call center, it would be helpful to have an estimate of how big that effect was in terms of the net number of callers gained. To measure the impact of the radio campaign, we need to estimate how many requests for family planning information the call center would have received in absence of the campaign. Single group ITS assumes that without the campaign, the pre-intervention trend would have continued. We estimated both the linear trend (which assumes a steady increase) and the logarithmic trend (which assumes a rapid increase, followed by a leveling off).

Based on the pre-intervention observed data, the linear trend was estimated as *y* = 2.8273 *x* + 544.13 and the logarithmic trend as *y* = 46.002 ln⁡( *x*) + 487.9, where *y* is the number of family planning related calls and *x* the time period (in months). Due to the random fluctuation and the two outliers, neither trend explained the variability in the number of calls. ^1^ After the start of the radio campaign, the level of the trend was notably higher, while the gradient was slightly negative (linear trend: *y* = –11.657 *x* + 1093.8; logarithmic trend: *y* = –12.14 ln⁡( *x*) + 1066.3).

**Figure 2.**
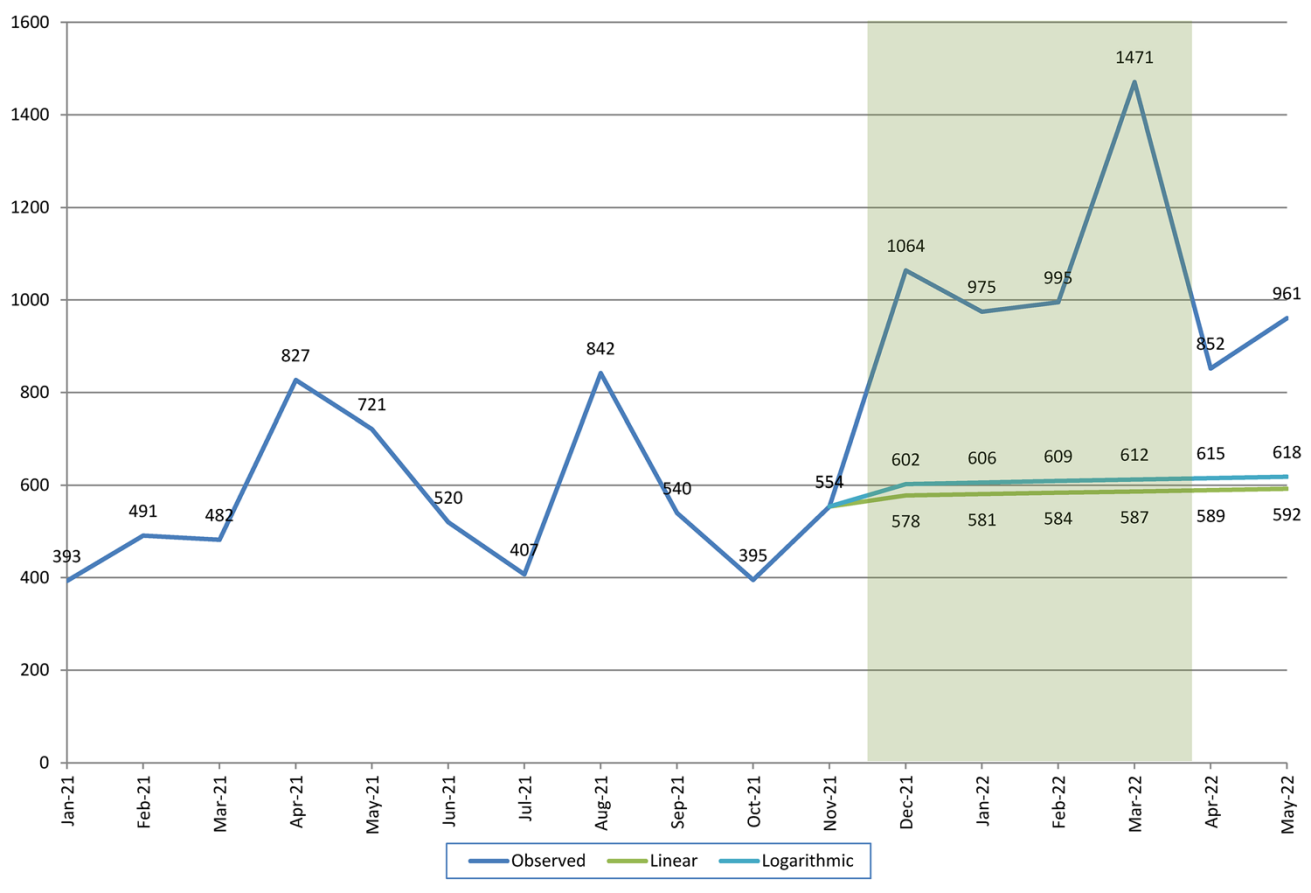
Actual and projected number of requests for family planning information. Note: Shaded area depicts the duration of the radio campaign.

Figure 2 shows the actual trend in the number of calls received during the entire study period, as well as the projected trend in absence of the radio campaign for the period from December 2021 through May 2022. The green line assumes that the trend is linear; the light blue line assumes that the trend is logarithmic. Consistent with our earlier observation that the monthly number of calls during the pre-campaign period was nearly constant (with the exception of the two peaks), both projections suggest that there would only have been a minimal increase in the number of requests for family planning information. Based on the linear projection, it is estimated that in absence of the radio campaign the monthly number of calls would have gradually increased from 554 in November (actual) to 592 in May. Based on the logarithmic projection, the increase would have been from 554 to 618.

Visual comparison of the projected and actual number of requests suggests that the radio campaign generated 400–800 additional information requests per month, for the duration of the campaign (December through March). Moreover, the data for April and May suggest that once the campaign had ended, the number of information requests remained considerably above the pre-campaign levels.

### Estimation of the campaign effect size

The difference between the actual number of calls received and the projected numbers is our estimate of the effect of the radio campaign on the number of callers requesting family planning information. Table 1 shows the actual number of calls received, the predicted number of calls using our two types of counterfactuals (ITS with linear and logarithmic projections), and the estimated number of calls gained for each model.

**Table 1.** Estimated number of family planning calls gained by the radio campaign (December 2021–May 2022).

	Observed number of calls	Predicted number of calls		Estimated effect (gain)	
	Total	Linear projection (ITS)	Logarithmic projection (ITS)	Linear projection (ITS)	Logarithmic projection (ITS)
12/21	1064	578	602	486	462
01/22	975	581	606	394	369
02/22	995	584	609	411	386
03/22	1471	587	612	884	859
04/22	852	589	615	263	237
05/22	961	592	618	369	343
Total	6318	3511	3664	2807	2654

Note: ITS Interrupted time series.

For the period from December 2021 through May 2022, the linear projection model estimates that there were 2,807 additional calls requesting family planning information, while the logarithmic projection puts the gain at 2,654 calls. While the true effect size of the radio campaign remains unknown, our analyses suggest it is likely be in the range of 2,600–2,800 additional requests for family planning information. To put that estimate in context, it implies that the radio campaign added the equivalent of the total number of requests for family planning information the call center would normally receive over a four to five month period. However, most of the gains occurred during the months while the radio campaign was active. As soon as the campaign ended, the gain in calls dropped to only 250–350 calls per month. In other words, the feedback data indicate that the radio campaign only led to a temporary increase in demand for family planning information.

## Discussion

Conventionally, public health programs–including family planning programs–have often used a fairly rigid intervention strategy. Decisions about the approach and intervention activities to use are typically made during the proposal development and/or design stage, and the agreed upon strategy is then implemented for the duration of the grant cycle, which may range from three to five years. End-of-project impact evaluations are then used to assess the extent to which the strategy was effective, and the lessons learned are incorporated into the design of follow-on programs. While end-of-project evaluations are necessary, the lengthy gap between such evaluations implies that the learning process is slow. Hence, there has been a push for programs to incorporate mechanisms that regularly provide feedback to program implementers about implementation problems, successes, and failures, so that appropriate programmatic adjustments can be made ( Harvard Kennedy School, 2014; Viswanath *et al.*, 2019). These feedback mechanisms also allow implementers to engage in short cycles of testing new activities, reflecting on lessons learned, and making programmatic adjustments, including discontinuing activities that do not live up to promise. As such, responsive feedback mechanisms go beyond traditional program monitoring, and can be considered to be a form of simple, continuous impact evaluations for program improvement.

The responsive feedback approach is based on the premises 1) that making regular course corrections in response to changing field circumstances will make programs more effective, and 2) that course corrections should be informed by regular feedback about what is and is not working as intended. Since most programs engage in routine data collection, it would be beneficial to explore whether more thorough analyses of those data can serve as a responsive feedback mechanism to inform program adjustments.

This study used analyzed routine data on the number of calls received by the Honey&Banana Connect call center in Nigeria to get regular feedback about the effectiveness of a radio jingle campaign for increasing the demand for family planning information. Effectiveness of the campaign was measured using a simple interrupted time series design, without a control group. The first phase of the ITS analysis consists of a simple review of the graphed results ( Jandoc *et al.*, 2015; Penfold & Zhang, 2013; Wagner *et al.*, 2002), which Honey&Banana was already doing as part of their program monitoring activities. Because the graphed results are easy to interpret, this already made it clear that the radio program triggered an increase in the number of calls received. The second phase of the ITS analysis consists of the creation of an artificial control group by projecting the pre-campaign trends in the number of calls received, which then enables quantification of the estimated number of calls that were added each month due to the radio campaign. Having an estimate of the number of new clients that were generated can be valuable for programs, because it helps clarify whether an intervention is performing up to expectations. In some cases, estimates of the number of new clients may also facilitate decision-making about staffing needs. It can also be valuable for reporting purposes in cases when grant proposals made explicit commitments about the number of new clients that would be generated.

Our results indicated that the radio campaign was generating only about 500 additional requests for family planning information per month, which was well below the number that H&B had hoped for, given the size and expense of the radio campaign. Because this feedback showed that the campaign was not living up to expectations, H&B made an informed decision to end the radio campaign prematurely. Because routine data collection continued after the campaign was ended, the ITS analysis also showed that the effect of the radio campaign was largely temporary. By using responsive feedback, H&B was able to shift resources to a different intervention activity that appeared more promising. Because small-scale campaigns offering e-vouchers for free contraceptive supplies had received a good response, H&B shifted resources freed up from the radio campaign toward the development of scaled-up e-voucher campaign. H&B will use the same analytical approach to obtain regular feedback on the effectiveness of this new e-voucher, reflect on the findings, and to make decisions about potential program adjustments.

Our study demonstrates that simple ITS analyses of routine data can be an invaluable tool for obtaining responsive feedback to inform programmatic adjustments. Basic ITS analyses–as illustrated here–can be conducted using only Excel spreadsheet software, and do not require extensive in-house research capacity. There are a number of simple ways to replicate the proposed approach to get more detailed programmatic information. Although our illustrative example only used one outcome measure (the number of calls received), it would be recommended to apply the same approach to all available outcome measures. For example, had the radio campaign resulted a drastic increase in the number of incoming calls, then the next step would be to use similar ITS analyses to access whether it also increased the number of callers who requested a referral to a family planning provider. Similarly, subject to data availability, it could be used to assess whether the radio intervention resulted in an increase in the number of clients who visited a network provider. Comparison of these findings would provide a simple way to assess to what extent an increase in the number of calls received by the call center translated into an increased number of referrals, and in turn into an increased number of family planning clients. If routine data exist for different population subgroups, the analysis can easily be replicated for each subgroup. For example, if data were collected on the number of male and female callers, or on the number of rural and urban callers, ITS analyses by subgroup could give additional insights about which subgroups were most responsive to the campaign.

The ITS design is particularly suitable for decision-making about social and mass media campaigns, as these typically have a clear starting date. Because many organizations already collect routine program data, using ITS analyses of routine data as a responsive feedback tool may be feasible without incurring additional data collection costs. Assuming data for relevant outcome measures are available, the design can be useful when the pre-intervention trend is well understood and when there are no known competing interventions during the study period. Hence, we recommend that public health organizations explore whether they can use ITS analyses of routine data as a responsive feedback mechanism for continuous program improvement.

## Consent

This study does not contain human subject data. The Tulane University Human Research Projection Office determined this study does not require IRB review and approval (Ref #2023-590)

## Data Availability

Harvard Dataverse: Monthly number of phone calls received by the Honey&Banana Connect family planning call center in Nigeria (January 2021 – December 2022). https://doi.org/10.7910/DVN/OMVLLB ( Olutola *et al.*, 2023). Data are available under the terms of the Creative Commons Zero "No rights reserved" data waiver (CC0 1.0 Public domain dedication).

## Footnotes

^1^ Based on the earlier observation that the pre-intervention included two outliers, we also calculated the pre-intervention trends both with and without the outliers. Both analyses produced similar results (not shown), but the omission of the outliers notably lowered the level of the trends. Although exclusion of the outliers improved the fit of trends, we have opted to only use the data that include the outliers because the small intervention activities that produced them are a more accurate representation of what would happen in absence of the radio campaign.
