# Supplementary material for: Using responsive feedback from routine monitoring data to guide course corrections for a family planning intervention in Nigeria
Source: Gates Open Res. 2023 Nov 16;7:75. Originally published 2023 May 19. [Version 2] doi: 10.12688/gatesopenres.14591.2 (PMC10821895; doi:10.12688/gatesopenres.14591.2)
Supplement: Peer review history

# Reviewer report: Reviewer response for version 2

**DOI:** 10.21956/gatesopenres.16468.r35508

**Published:** 2024-01-27

**Authors:** Diwakar Mohan

**Affiliations:** 1 Department of International Health, Johns Hopkins University Bloomberg School of Public Health, Baltimore, MD, USA

The comments raised have been addressed by the authors.

Is the work clearly and accurately presented and does it cite the current literature?

Partly

If applicable, is the statistical analysis and its interpretation appropriate?

Partly

Are all the source data underlying the results available to ensure full reproducibility?

Yes

Is the study design appropriate and is the work technically sound?

Partly

Are the conclusions drawn adequately supported by the results?

Partly

Are sufficient details of methods and analysis provided to allow replication by others?

Partly

Reviewer Expertise:

quantitative research methods

I confirm that I have read this submission and believe that I have an appropriate level of expertise to confirm that it is of an acceptable scientific standard.

# Reviewer report: Reviewer response for version 1

**DOI:** 10.21956/gatesopenres.15907.r35112

**Published:** 2023-10-27

**Authors:** Diwakar Mohan

**Affiliations:** 1 Department of International Health, Johns Hopkins University Bloomberg School of Public Health, Baltimore, MD, USA

The manuscript is well written, free of errors and does not need English language editing.

 I would like to focus my feedback on 2 broad areas.

**1. Need and relevance of ITS for monitoring:**

- a. Monitoring requires near live (relatively) time data collection and analysis - timely and flexible enough to allow for programmatic changes. This has been covered as part of the statistical methods designed for quality improvement studies in health care and has extensive literature backing it up. The authors have not made a clear case for the advantage of the ITS methodology over these pre-existing methods.
   
  - Brady *et al.* (2018 ^1^).
  - McQuillan *et al.* (2016 ^2^).
- b. By the time one has enough sufficient data points to have a reasonable inference, it may be too late for course corrections. While in this present instance, the impact (referrals to and use of FP) may be logically implied from the output (number of calls), this may not always be the case in all instances. For example, the quality rather than the quantity of the output may be important. To use the present scenario as an example, if the conversion rate of the calls does not keep to a minimum the increase in calls is nothing more than a cost without the resultant benefit of FP use. Those monitoring the calls may be lulled into a misplaced sense of complacency by looking at the number of calls alone.
- c. Accounting for lag – While the present example allowed for no lag due to its nature, this may not be the usual case in most other interventions/programs. When the effect is insidious and can take more time than the time points used for the data, the effects may be difficult to separate/assess or too late.
- d. Nature of the outcome – While a continuous numerical outcome provides the maximum efficiency for detection of changes, it may not always be possible to rely on one being available and relevant. Offsets could account for the ‘denominator’ but may be more difficult to get.

**2. Methodological considerations for ITS:**

 The present ITS work seems to work as an evaluation of the radio campaign to increase callers to the DKT helpline. Some suggestions to improve the analysis presented are provided below:

- a. Description of analyses – The present study has used the inbuilt excel function to generate separate regression lines fitting the points before and after based on Ordinary Least Squares regression (I assume). Conventionally, we have used segmented regressions, ARIMA models or change point analysis for ITS (this is my first experience with ITS on excel). The present does appear to capture the essence of the segmented regression approach though the power is very much reduced and has higher susceptibility to outliers.
- b. Results – traditionally ITS effects have been reported in terms of change in level, and change in trend. With the present study, it is obvious that there is change in level (difference in intercepts of the two lines), but the changes in trend are difficult to interpret. The lack of confidence intervals appears to make any interpretations of significance quite challenging.
- c. Treatment of outliers – the authors have reported identifying outliers. The inability to separate the effects due to such values is one of the important reasons that the ITS may not serve well as a monitoring device.
- d. Reporting accuracy of prediction models is usually done using metrics such as RMSE. It is difficult to bench mark the accuracy using such tables. Moreover, how are program implementers expected to use such predictions?

Is the work clearly and accurately presented and does it cite the current literature?

Partly

If applicable, is the statistical analysis and its interpretation appropriate?

Partly

Are all the source data underlying the results available to ensure full reproducibility?

Yes

Is the study design appropriate and is the work technically sound?

Partly

Are the conclusions drawn adequately supported by the results?

Partly

Are sufficient details of methods and analysis provided to allow replication by others?

Partly

Reviewer Expertise:

quantitative research methods

I confirm that I have read this submission and believe that I have an appropriate level of expertise to confirm that it is of an acceptable scientific standard, however I have significant reservations, as outlined above.

## References

[1] : Quality Improvement Feature Series Article 2: Displaying and Analyzing Quality Improvement Data. *J Pediatric Infect Dis Soc* .2018;7(2) : 10.1093/jpids/pix077 100-103 10.1093/jpids/pix077 29040644

[2] : How to Measure and Interpret Quality Improvement Data. *Clin J Am Soc Nephrol* .2016;11(5) : 10.2215/CJN.11511015 908-914 10.2215/CJN.11511015 27016496 PMC4858492

# Response

**Published:** 2023-11-12

**Authors:** Dominique Meekers

a. Monitoring requires near live (relatively) time data collection and analysis - timely and flexible enough to allow for programmatic changes. This has been covered as part of the statistical methods designed for quality improvement studies in health care and has extensive literature backing it up. The authors have not made a clear case for the advantage of the ITS methodology over these pre-existing methods.

**Reply:** The run chart and control chart methodologies that are described in Brady and McQuillan are clearly very useful for quality improvement studies. However, our study is not a quality improvement study. The objective is not to track and improve the quality of the call center agents, nor is it to track and improve the quality of the radio broadcasts. Rather, the objective is to estimate the increase in demand (in our case, the total number of clients that were gained because of the radio broadcast). Neither of the cited techniques do that.  Having an estimate of the number of clients gained can be valuable for several reasons, for example when implementers have made promises to their donor about the number of new clients that will be achieved (which is common). Knowing the number of clients gained can also help program implementers make decisions about staffing needs etc. We have clarified this in the discussion section.

 b. By the time one has enough sufficient data points to have a reasonable inference, it may be too late for course corrections. While in this present instance, the impact (referrals to and use of FP) may be logically implied from the output (number of calls), this may not always be the case in all instances. For example, the quality rather than the quantity of the output may be important. To use the present scenario as an example, if the conversion rate of the calls does not keep to a minimum the increase in calls is nothing more than a cost without the resultant benefit of FP use. Those monitoring the calls may be lulled into a misplaced sense of complacency by looking at the number of calls alone.

**Reply:** The reviewer is correct that an increase in the number of calls does not necessarily lead to improvements in family planning use. However, the aim of our paper is to advocate for simple further analyses of routine monitoring data to guide program improvement. We do not advocate restricting such analyses to a single outcome measure. In our specific example, the main objective of the radio jingle was to promote use of the call center. Hence, the number of calls received is a relevant outcome. Nevertheless, as noted by the reviewer, the expectation is obviously that these calls will translate into increased family planning use.  Had the campaign yielded a more substantial increase in the number of call (and had not been prematurely terminated), the logical next step would have been to simply replicate the analysis for other outcome measures that are from available routine data collection. For example, that may include the monthly number of referrals to a family planning provider, or the monthly number of CYPs distributed by network family planning providers. As noted by the other reviewer, if routine data exist by subgroup, replicating the analysis for different subgroups (e.g., males/females, rural/urban) may also provide helpful programmatic information. We have emphasized in the discussion section that it is important to examine all relevant outcome measures, provided such data are available.

 c. Accounting for lag – While the present example allowed for no lag due to its nature, this may not be the usual case in most other interventions/programs. When the effect is insidious and can take more time than the time points used for the data, the effects may be difficult to separate/assess or too late.

**Reply:** It is certainly true that for some interventions, it may take some time before it yields any effect (i.e., there is a lag), and that this would therefore require a longer data series. Of course, that would also be the case for more sophisticated analytical methods. Given that the proposed approach uses only existing routine data and does not require any time-consuming analyses, we contend that there is little risk in trying.

 d. Nature of the outcome – While a continuous numerical outcome provides the maximum efficiency for detection of changes, it may not always be possible to rely on one being available and relevant. Offsets could account for the ‘denominator’ but may be more difficult to get.

**Reply:** Agreed. The type of routine data that are being collected will vary across projects.  As with any research project, will be important to ensure that the outcome measures used are relevant. In absence of relevant data, there will be nothing to analyze.

 The present ITS work seems to work as an evaluation of the radio campaign to increase callers to the DKT helpline. Some suggestions to improve the analysis presented are provided below:

 a. Description of analyses – The present study has used the inbuilt excel function to generate separate regression lines fitting the points before and after based on Ordinary Least Squares regression (I assume). Conventionally, we have used segmented regressions, ARIMA models or change point analysis for ITS (this is my first experience with ITS on excel). The present does appear to capture the essence of the segmented regression approach though the power is very much reduced and has higher susceptibility to outliers.

**Reply:** The reviewer is correct that more powerful analytical techniques exist (e.g., ARIMA models). However, numerous implementing organizations would not normally conduct or commission such analyses. We hoped to make the case that the simplicity of the approach and the use of existing data implies that it can be used by organizations that have very minimal research capacity or resources, and to illustrate that the approach -- despite its simplicity -- can still provide helpful information to guide programs. We have revised both the abstract and introduction sections to clarify this.

 b. Results – traditionally ITS effects have been reported in terms of change in level, and change in trend. With the present study, it is obvious that there is change in level (difference in intercepts of the two lines), but the changes in trend are difficult to interpret. The lack of confidence intervals appears to make any interpretations of significance quite challenging.

**Reply:** Confidence intervals and/or levels of statistical significance are important when analyzing sample data. However, it would be unusual for routine data collection to be based on a sample. Routine data collection normally pertains to all customers or users, which implies the data are in fact a census. Hence, there is no concern that one may have selected an unusual sample. However, if one wanted to have a range of plausible estimates of the outcomes in absence of the intervention, one could conduct some kind of sensitivity analysis, by varying the assumptions for the projected trend. For example, in addition to the assumption that the pre-intervention trend would continue, one could conduct projections that assume there would have been, say a 5% increase in the slope. Since we are particularly interested in simple approaches that can be applied by organizations that have limited research capacity, we have opted not to address that.

 c. Treatment of outliers – the authors have reported identifying outliers. The inability to separate the effects due to such values is one of the important reasons that the ITS may not serve well as a monitoring device.

**Reply:** The reviewer is correct that there may be instances in which the proposed technique will not be suitable. In any study that detects outliers, it will be important to try and identify the cause of those outliers. If the pre-intervention data pattern is poorly understood, then it will be hard to make the case this pattern would have continued without the intervention. We have emphasized this in the “study design” section. (The option to run the analysis with the outliers removed, as is done by some other authors is also discussed in paragraph 1 of the results, and in footnote 1).

 d. Reporting accuracy of prediction models is usually done using metrics such as RMSE. It is difficult to bench mark the accuracy using such tables. Moreover, how are program implementers expected to use such predictions?

**Reply:** As the reviewer probably suspected, the only goodness of fit metric that Excel provides is the R ^2^. But no matter which metric is used, the true post-intervention counterfactual is always unknown. Implementing organizations that are interested in metrics to benchmark the accuracy of the predictions would be better off using the more advanced analytical techniques mentioned earlier (e.g., ARIMA).  However, numerous organizations have very limited research capacity. Such organizations are unlikely to make programmatic decisions based on statistical analyses or metrics that they are not familiar with (and that may intimidate them). We contend that they will benefit more from simpler methods such as the one described. We have clarified our aim in the abstract and introduction.

# Reviewer report: Reviewer response for version 1

**DOI:** 10.21956/gatesopenres.15907.r34025

**Published:** 2023-08-16

**Authors:** Susan Igras

**Affiliations:** 1 Institute for Reproductive Health, Center for Child and Human Development, Georgetown University, Washington, USA

This research article focuses on applying a research/outcome evaluation method, interrupted time series (ITS) analysis of routine data, to guide mid-program decision-making on a new FP-promotion strategy that encourages potential family planning users to seek additional information on contraceptive methods and where to access them in Nigeria. The data (outcome indicator) used existed in call records from a call-in center program. The question was whether the new FP-promotion call-in strategy yielded enough new callers to merit its continuation. The well-written article articulates and promotes an innovative application of ITS as a tool for responsive feedback. So interesting! It develops conceptually the ‘why’ and provides practical guidance on ‘how’ for others interested in using ITS as a responsive feedback analytic tool. Although the article is not positioned as such, the utility of ITS is also helpful for those following adaptive management approaches, e.g., as promoted within the evaluation consolidation site 'Better Evaluation', USAID's Learning Lab and ODI's global learning for adaptive management initiative, which also recognize systems complexities and the need to assess and adjust strategies throughout a program. NGO implementers as well as scholars and donors of health and social development programs are increasingly testing and using such approaches.

 My one methodological comment is that it would have been interesting to continue to collect data for six months after the closing of the promotion campaign (versus one month in this study) to get a better understanding and confirmation of the trend data, given the campaign yielded more callers and at a higher monthly rate than in the 12 months before the campaign, meaning that the campaign could have had a more additive effect than recorded/projected. In the discussion, I also would have liked a bit more reflection by authors on future applications using ITS as a responsive feedback tool. Do other linked data exist to answer other questions about the costs-benefits of similar campaigns, e.g., in the Nigeria example what other data are routinely collected for each call, such as a caller's gender or age, that could be included in a future analyses? Some FP promotion programs have found, for example, that men are more receptive to radio messaging than women (Kim *et al.* 2021); what are the program tradeoffs using a gender lens when deciding to continue or not an approach?

 Overall, this article is a solid contribution to thinking about new ways to re-purpose routinely collected quantitative data that have more uses than initially intended and helps programs continually improve their efforts and effectiveness.

References:

1. https://www.betterevaluation.org/methods-approaches/themes/monitoring-evaluation-support-adaptive-management
2. https://usaidlearninglab.org/community/blog/what-adaptive-management#:~:text=Adaptive%20management%20is%20defined%20in,goals%20in%20response%20to%20changes.
3. https://odi.org/en/about/our-work/the-global-learning-for-adaptive-management-initiative-glam/

Is the work clearly and accurately presented and does it cite the current literature?

Yes

If applicable, is the statistical analysis and its interpretation appropriate?

Yes

Are all the source data underlying the results available to ensure full reproducibility?

No

Is the study design appropriate and is the work technically sound?

Yes

Are the conclusions drawn adequately supported by the results?

Yes

Are sufficient details of methods and analysis provided to allow replication by others?

Yes

Reviewer Expertise:

Social and normative determinants of family planning; realism and realist evaluation to understand how programs works in complex contexts; MEL of SBC initiatives.

I confirm that I have read this submission and believe that I have an appropriate level of expertise to confirm that it is of an acceptable scientific standard.

## References

[1] : The power of women's and men's Social Networks to catalyse normative and behavioural change: evaluation of an intervention addressing Unmet need for Family Planning in Benin. *BMC Public Health* .2022;22(1) : 10.1186/s12889-022-12681-4 672 10.1186/s12889-022-12681-4 35392862 PMC8988370
